# *trans*-Bis[1-(2-anilino-2-oxoeth­yl)-3-benzyl-1*H*-imidazol-2-yl]palladium(II) methanol disolvate

**DOI:** 10.1107/S1600536811007768

**Published:** 2011-03-19

**Authors:** Hon Man Lee, Yu-Chuan Chang

**Affiliations:** a National Changhua University of Education, Department of Chemistry, Changhua, Taiwan 50058

## Abstract

In the title compound, [Pd(C_18_H_16_N_3_O)_2_]·2CH_3_OH, the Pd^II^ atom is located on a crystallographic inversion center. It has a square-planar coordination geometry, with the two bidentate ligands coordinated in a *trans* fashion *via* the carbene C atom and the amido N atoms. The methanol solvent mol­ecules form O—H⋯O hydrogen bonds with the complex. Additional non-classical inter­molecular C—H⋯O hydrogen bonds link the complexes into a two-dimensional network parallel to (001).

## Related literature

Palladium complexes with multidentate ligands containing *N*-heterocyclic carbene and anionic amidate functionalities attract inter­est because of their effectiveness in catalysing C—C coupling reactions, see: Liao *et al.* (2007 ▶); Sakaguchi *et al.* (2008 ▶).
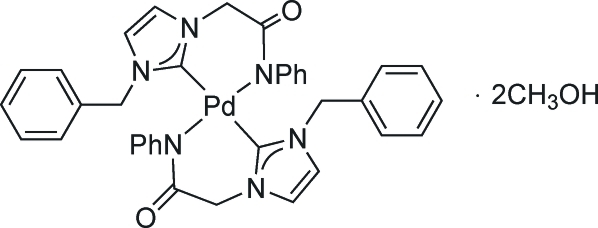


## Experimental

### 

#### Crystal data

[Pd(C_18_H_16_N_3_O)_2_]·2CH_4_O
*M* *_r_* = 751.16
Orthorhombic, 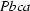

*a* = 17.822 (2) Å
*b* = 9.0616 (11) Å
*c* = 21.473 (3) Å
*V* = 3467.8 (7) Å^3^
*Z* = 4
Mo *K*α radiation
μ = 0.59 mm^−1^
*T* = 150 K
0.39 × 0.09 × 0.08 mm

#### Data collection

Bruker SMART APEXII diffractometer
Absorption correction: multi-scan (*SADABS*; Sheldrick, 2003 ▶) *T*_min_ = 0.804, *T*_max_ = 0.955
45827 measured reflections
4451 independent reflections
2695 reflections with *I* > 2σ
*R*_int_ = 0.080

#### Refinement

*R*[*F* ^2^ > 2σ(*F* ^2^)] = 0.032
*wR*(*F* ^2^) = 0.084
*S* = 1.00
4451 reflections
224 parameters
H-atom parameters constrained
Δρ_max_ = 0.39 e Å^−3^
Δρ_min_ = −0.67 e Å^−3^

### 

Data collection: *APEX2* (Bruker, 2007 ▶); cell refinement: *SAINT* (Bruker, 2007 ▶); data reduction: *SAINT*; program(s) used to solve structure: *SHELXTL* (Sheldrick, 2008 ▶); program(s) used to refine structure: *SHELXTL*; molecular graphics: *SHELXTL*; software used to prepare material for publication: *DIAMOND* (Brandenburg, 2006 ▶).

## Supplementary Material

Crystal structure: contains datablocks I, iupac. DOI: 10.1107/S1600536811007768/fj2398sup1.cif

Structure factors: contains datablocks I. DOI: 10.1107/S1600536811007768/fj2398Isup2.hkl

Additional supplementary materials:  crystallographic information; 3D view; checkCIF report

## Figures and Tables

**Table 1:** Hydrogen-bond geometry (Å, °)

*D*—H⋯*A*	*D*—H	H⋯*A*	*D*⋯*A*	*D*—H⋯*A*
O2—H1⋯O1^i^	0.92	1.81	2.727 (3)	172
C2—H2⋯O1^ii^	0.95	2.36	3.232 (3)	152
C18—H18⋯O1	0.95	2.32	2.842 (3)	114

Symmetry codes: (i) 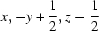; (ii) 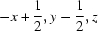.

**Fig. 1.**
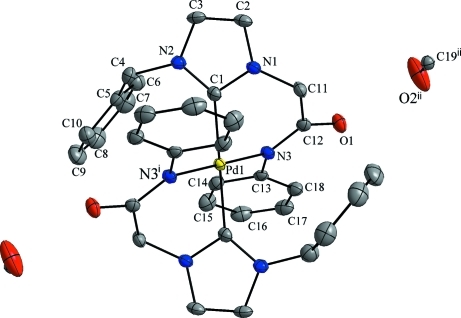
The structure of the title complex, showing 50% displacement ellipsoids. H atoms are excluded for clarity. [Symmetry code: (i) 1 - x, 1 - y, 2 - z. (ii) x, 1/2 - y, 1/2 + z.]

**Fig. 2.**
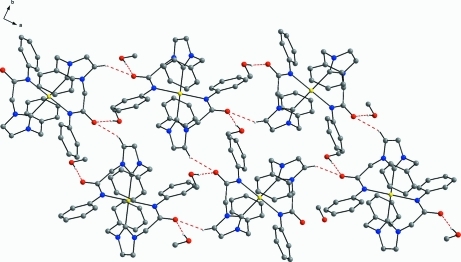
A view of the crystal packing along the c axis, displaying the hydrogen bonds as dashed lines.

## supplementary crystallographic information

### Comment

Palladium complexes with multidentate ligands containing *N*-heterocyclic carbene and anionic amidate functionalities attract interest because of their effectiveness in catalyzing C—C coupling reactions (Liao *et al.*, 2007 and Sakaguchi *et al.*, 2008). The crystal structure of the title compound consists of such palladium carbene complex with two solvated methanol molecules incorporated. The structure of a DMSO solvate of the same *trans* compound, C_36_H_32_N_6_O_2_Pd.4C_2_H_6_SO, was reported by us previously (Liao *et al.*, 2007)

The palladium atom adopts square coordination geometry with two *trans* coordinated bidentate ligands. The structure of the *cis* isomer, C_36_H_32_N_6_O_2_Pd.2CH_3_OH, was also reported earlier (Liao *et al.*, 2007). A comparison of the geometric parameters of the *trans* and *cis* isomers shows that the Pd—C bond distance in the *trans* isomer is longer than that in the *cis* isomer [2.014 (2) *vs*. 1.966 (2) Å]. Contrastingly, the Pd—N bond distance is shorter in the *trans* isomer [2.051 (2) *vs*. 2.087 (1) Å].

### Experimental

The title compound was prepared according to the literature procedure (Liao *et al.*, 2007). Colorless crystals suitable for X-ray diffraction analysis were grown by slow evaporation of a methanol solution containing the compound.

### Refinement

All the H atoms were positioned geometrically and refined as riding atoms, with C_aryl_—H = 0.95, C_methylene_ —H = 0.99, and C_methyl_ —H = 0.98 Å while *U*_iso_(H) = 1.2*U*_eq_(C_methine_), *U*_iso_(H) = 1.2*U*_eq_(C_methylene_), and *U*_iso_(H) = 1.5 *U*_eq_ (C_methyl_). H1 bound to oxygen was found in the difference Fourier map, not refined and with *U*_iso_(H) = 1.2*U*_eq_(O).

### Crystal data

**Table:**

[Pd(C_18_H_16_N_3_O)_2_]·2CH_4_O	*F*(000) = 1552
*M**_r_* = 751.16	*D*_x_ = 1.439 Mg m^−^^3^
Orthorhombic, *P**b**c**a*	Mo *K*α radiation, λ = 0.71073 Å
Hall symbol: -P 2ac 2ab	Cell parameters from 3616 reflections
*a* = 17.822 (2) Å	θ = 2.7–22.4°
*b* = 9.0616 (11) Å	µ = 0.59 mm^−^^1^
*c* = 21.473 (3) Å	*T* = 150 K
*V* = 3467.8 (7) Å^3^	Parallelpiped, white
*Z* = 4	0.39 × 0.09 × 0.08 mm

### Data collection

**Table:**

Bruker SMART APEXII diffractometer	4451 independent reflections
Radiation source: fine-focus sealed tube	2695 reflections with *I* > 2σ
graphite	*R*_int_ = 0.080
ω scans	θ_max_ = 28.7°, θ_min_ = 1.9°
Absorption correction: multi-scan (*SADABS*; Sheldrick, 2003)	*h* = −21→23
*T*_min_ = 0.804, *T*_max_ = 0.955	*k* = −12→12
45827 measured reflections	*l* = −28→28

### Refinement

**Table:**

Refinement on *F*^2^	Primary atom site location: structure-invariant direct methods
Least-squares matrix: full	Secondary atom site location: difference Fourier map
*R*[*F*^2^ > 2σ(*F*^2^)] = 0.032	Hydrogen site location: inferred from neighbouring sites
*wR*(*F*^2^) = 0.084	H-atom parameters constrained
*S* = 1.00	*w* = 1/[σ^2^(*F*_o_^2^) + (0.0273*P*)^2^ + 3.0876*P*] where *P* = (*F*_o_^2^ + 2*F*_c_^2^)/3
4451 reflections	(Δ/σ)_max_ = 0.001
224 parameters	Δρ_max_ = 0.39 e Å^−^^3^
0 restraints	Δρ_min_ = −0.67 e Å^−^^3^

### Special details

**Table:**

Geometry. All e.s.d.'s (except the e.s.d. in the dihedral angle between two l.s. planes) are estimated using the full covariance matrix. The cell e.s.d.'s are taken into account individually in the estimation of e.s.d.'s in distances, angles and torsion angles; correlations between e.s.d.'s in cell parameters are only used when they are defined by crystal symmetry. An approximate (isotropic) treatment of cell e.s.d.'s is used for estimating e.s.d.'s involving l.s. planes.
Refinement. Refinement of *F*^2^ against ALL reflections. The weighted *R*-factor *wR* and goodness of fit *S* are based on *F*^2^, conventional *R*-factors *R* are based on *F*, with *F* set to zero for negative *F*^2^. The threshold expression of *F*^2^ > σ(*F*^2^) is used only for calculating *R*-factors(gt) *etc*. and is not relevant to the choice of reflections for refinement. *R*-factors based on *F*^2^ are statistically about twice as large as those based on *F*, and *R*- factors based on ALL data will be even larger.

### Fractional atomic coordinates and isotropic or equivalent isotropic displacement parameters (Å^2^)

**Table:**

	*x*	*y*	*z*	*U*_iso_*/*U*_eq_	
C1	0.46400 (13)	0.2896 (2)	0.99551 (11)	0.0171 (5)	
C2	0.38152 (15)	0.1075 (3)	1.01712 (12)	0.0228 (6)	
H2	0.3418	0.0545	1.0363	0.027*	
C3	0.42383 (14)	0.0630 (3)	0.96912 (13)	0.0226 (6)	
H3	0.4198	−0.0284	0.9477	0.027*	
C4	0.52739 (16)	0.1767 (3)	0.90363 (11)	0.0219 (5)	
H4A	0.5339	0.0748	0.8879	0.026*	
H4B	0.5769	0.2124	0.9181	0.026*	
C5	0.49996 (16)	0.2745 (3)	0.85115 (11)	0.0217 (5)	
C6	0.42429 (16)	0.2778 (3)	0.83425 (12)	0.0280 (6)	
H6	0.3890	0.2191	0.8563	0.034*	
C7	0.40060 (18)	0.3664 (3)	0.78546 (13)	0.0350 (7)	
H7	0.3491	0.3676	0.7740	0.042*	
C8	0.45124 (19)	0.4528 (3)	0.75345 (14)	0.0385 (8)	
H8	0.4347	0.5133	0.7200	0.046*	
C9	0.5260 (2)	0.4511 (4)	0.77007 (14)	0.0389 (8)	
H9	0.5609	0.5111	0.7482	0.047*	
C10	0.55057 (17)	0.3619 (3)	0.81869 (13)	0.0300 (6)	
H10	0.6022	0.3608	0.8297	0.036*	
C11	0.37610 (15)	0.3458 (3)	1.07994 (11)	0.0211 (6)	
H11A	0.4153	0.3697	1.1110	0.025*	
H11B	0.3347	0.2947	1.1019	0.025*	
C12	0.34608 (14)	0.4900 (3)	1.05140 (11)	0.0181 (5)	
C13	0.35913 (13)	0.6589 (2)	0.96519 (11)	0.0165 (5)	
C14	0.39053 (15)	0.6620 (3)	0.90513 (12)	0.0222 (6)	
H14	0.4311	0.5979	0.8955	0.027*	
C15	0.36355 (16)	0.7567 (3)	0.85971 (12)	0.0286 (6)	
H15	0.3859	0.7576	0.8195	0.034*	
C16	0.30402 (16)	0.8504 (3)	0.87279 (13)	0.0296 (6)	
H16	0.2846	0.9140	0.8415	0.036*	
C17	0.27323 (16)	0.8499 (3)	0.93189 (13)	0.0279 (6)	
H17	0.2327	0.9145	0.9411	0.033*	
C18	0.30045 (14)	0.7565 (3)	0.97816 (13)	0.0229 (5)	
H18	0.2791	0.7592	1.0187	0.027*	
C19	0.2385 (2)	0.1346 (5)	0.70995 (15)	0.0574 (10)	
H19A	0.2522	0.2332	0.6950	0.086*	
H19B	0.2842	0.0770	0.7175	0.086*	
H19C	0.2100	0.1434	0.7488	0.086*	
N1	0.40758 (11)	0.2469 (2)	1.03309 (9)	0.0182 (4)	
N2	0.47490 (12)	0.1753 (2)	0.95629 (9)	0.0184 (4)	
N3	0.38849 (11)	0.5529 (2)	1.00742 (9)	0.0174 (4)	
O1	0.28388 (10)	0.5348 (2)	1.07073 (8)	0.0254 (4)	
Pd1	0.5000	0.5000	1.0000	0.01396 (7)	
O2	0.19462 (14)	0.0636 (3)	0.66513 (12)	0.0650 (8)	
H1	0.2245	0.0384	0.6316	0.078*	

### Atomic displacement parameters (Å^2^)

**Table:**

	*U*^11^	*U*^22^	*U*^33^	*U*^12^	*U*^13^	*U*^23^
C1	0.0130 (12)	0.0172 (11)	0.0210 (11)	0.0014 (9)	0.0003 (11)	0.0020 (10)
C2	0.0202 (13)	0.0146 (12)	0.0335 (14)	−0.0036 (11)	0.0002 (11)	0.0052 (10)
C3	0.0199 (14)	0.0127 (11)	0.0351 (15)	−0.0028 (11)	−0.0030 (12)	−0.0006 (11)
C4	0.0214 (13)	0.0206 (12)	0.0236 (13)	0.0043 (11)	0.0040 (11)	−0.0034 (11)
C5	0.0279 (14)	0.0172 (11)	0.0200 (11)	0.0047 (12)	0.0003 (12)	−0.0033 (9)
C6	0.0303 (16)	0.0269 (15)	0.0268 (14)	−0.0002 (12)	−0.0007 (12)	−0.0009 (12)
C7	0.0358 (18)	0.0377 (17)	0.0314 (15)	0.0086 (14)	−0.0057 (13)	0.0001 (13)
C8	0.051 (2)	0.0376 (16)	0.0266 (15)	0.0098 (16)	−0.0003 (14)	0.0056 (13)
C9	0.049 (2)	0.0359 (16)	0.0319 (16)	0.0023 (15)	0.0117 (15)	0.0086 (14)
C10	0.0300 (16)	0.0317 (15)	0.0282 (14)	−0.0001 (13)	0.0055 (12)	0.0008 (12)
C11	0.0200 (14)	0.0221 (13)	0.0212 (12)	0.0009 (11)	0.0073 (11)	0.0041 (10)
C12	0.0157 (12)	0.0183 (12)	0.0203 (11)	−0.0023 (11)	−0.0004 (9)	−0.0031 (11)
C13	0.0125 (12)	0.0133 (11)	0.0237 (13)	−0.0028 (9)	−0.0009 (10)	−0.0022 (10)
C14	0.0209 (14)	0.0197 (13)	0.0258 (13)	0.0026 (11)	−0.0010 (11)	−0.0041 (11)
C15	0.0343 (17)	0.0304 (15)	0.0212 (13)	0.0010 (13)	−0.0057 (12)	−0.0004 (11)
C16	0.0339 (17)	0.0219 (14)	0.0331 (15)	0.0031 (12)	−0.0119 (13)	0.0043 (12)
C17	0.0205 (15)	0.0213 (13)	0.0418 (16)	0.0038 (11)	−0.0023 (12)	0.0013 (12)
C18	0.0191 (13)	0.0178 (12)	0.0317 (13)	0.0013 (11)	0.0025 (11)	0.0006 (11)
C19	0.053 (2)	0.082 (3)	0.0367 (18)	−0.011 (2)	0.0133 (17)	−0.0195 (19)
N1	0.0152 (10)	0.0156 (10)	0.0238 (11)	−0.0014 (9)	0.0028 (9)	0.0021 (8)
N2	0.0186 (11)	0.0153 (10)	0.0213 (10)	−0.0001 (8)	0.0011 (9)	0.0023 (9)
N3	0.0112 (10)	0.0166 (8)	0.0245 (11)	−0.0006 (8)	0.0019 (8)	0.0005 (8)
O1	0.0169 (10)	0.0312 (10)	0.0279 (9)	0.0047 (8)	0.0069 (8)	0.0044 (8)
Pd1	0.01066 (11)	0.01262 (11)	0.01860 (11)	−0.00022 (11)	0.00200 (11)	0.00015 (11)
O2	0.0374 (15)	0.104 (2)	0.0534 (15)	−0.0128 (15)	0.0180 (12)	−0.0321 (15)

### Geometric parameters (Å, °)

**Table:**

C1—N1	1.346 (3)	C11—H11A	0.9900
C1—N2	1.349 (3)	C11—H11B	0.9900
C1—Pd1	2.014 (2)	C12—O1	1.252 (3)
C2—C3	1.339 (4)	C12—N3	1.337 (3)
C2—N1	1.388 (3)	C13—C18	1.398 (3)
C2—H2	0.9500	C13—C14	1.406 (3)
C3—N2	1.393 (3)	C13—N3	1.421 (3)
C3—H3	0.9500	C14—C15	1.385 (4)
C4—N2	1.468 (3)	C14—H14	0.9500
C4—C5	1.515 (3)	C15—C16	1.387 (4)
C4—H4A	0.9900	C15—H15	0.9500
C4—H4B	0.9900	C16—C17	1.383 (4)
C5—C10	1.388 (4)	C16—H16	0.9500
C5—C6	1.397 (4)	C17—C18	1.392 (4)
C6—C7	1.386 (4)	C17—H17	0.9500
C6—H6	0.9500	C18—H18	0.9500
C7—C8	1.378 (4)	C19—O2	1.397 (4)
C7—H7	0.9500	C19—H19A	0.9800
C8—C9	1.379 (5)	C19—H19B	0.9800
C8—H8	0.9500	C19—H19C	0.9800
C9—C10	1.391 (4)	N3—Pd1	2.050 (2)
C9—H9	0.9500	Pd1—C1^i^	2.014 (2)
C10—H10	0.9500	Pd1—N3^i^	2.051 (2)
C11—N1	1.460 (3)	O2—H1	0.9244
C11—C12	1.540 (3)		
			
N1—C1—N2	105.1 (2)	N3—C12—C11	116.5 (2)
N1—C1—Pd1	118.80 (17)	C18—C13—C14	117.9 (2)
N2—C1—Pd1	135.30 (18)	C18—C13—N3	125.2 (2)
C3—C2—N1	106.0 (2)	C14—C13—N3	116.9 (2)
C3—C2—H2	127.0	C15—C14—C13	121.3 (2)
N1—C2—H2	127.0	C15—C14—H14	119.3
C2—C3—N2	107.5 (2)	C13—C14—H14	119.3
C2—C3—H3	126.3	C14—C15—C16	120.1 (3)
N2—C3—H3	126.3	C14—C15—H15	119.9
N2—C4—C5	111.9 (2)	C16—C15—H15	119.9
N2—C4—H4A	109.2	C17—C16—C15	119.2 (3)
C5—C4—H4A	109.2	C17—C16—H16	120.4
N2—C4—H4B	109.2	C15—C16—H16	120.4
C5—C4—H4B	109.2	C16—C17—C18	121.2 (3)
H4A—C4—H4B	107.9	C16—C17—H17	119.4
C10—C5—C6	119.0 (2)	C18—C17—H17	119.4
C10—C5—C4	119.9 (3)	C17—C18—C13	120.2 (3)
C6—C5—C4	121.1 (2)	C17—C18—H18	119.9
C7—C6—C5	120.2 (3)	C13—C18—H18	119.9
C7—C6—H6	119.9	O2—C19—H19A	109.5
C5—C6—H6	119.9	O2—C19—H19B	109.5
C8—C7—C6	120.4 (3)	H19A—C19—H19B	109.5
C8—C7—H7	119.8	O2—C19—H19C	109.5
C6—C7—H7	119.8	H19A—C19—H19C	109.5
C7—C8—C9	119.8 (3)	H19B—C19—H19C	109.5
C7—C8—H8	120.1	C1—N1—C2	111.3 (2)
C9—C8—H8	120.1	C1—N1—C11	121.6 (2)
C8—C9—C10	120.3 (3)	C2—N1—C11	126.9 (2)
C8—C9—H9	119.8	C1—N2—C3	110.1 (2)
C10—C9—H9	119.8	C1—N2—C4	124.5 (2)
C5—C10—C9	120.2 (3)	C3—N2—C4	125.1 (2)
C5—C10—H10	119.9	C12—N3—C13	122.0 (2)
C9—C10—H10	119.9	C12—N3—Pd1	120.21 (16)
N1—C11—C12	112.36 (19)	C13—N3—Pd1	117.74 (15)
N1—C11—H11A	109.1	C1^i^—Pd1—C1	179.999 (1)
C12—C11—H11A	109.1	C1^i^—Pd1—N3	94.81 (9)
N1—C11—H11B	109.1	C1—Pd1—N3	85.19 (9)
C12—C11—H11B	109.1	C1^i^—Pd1—N3^i^	85.19 (9)
H11A—C11—H11B	107.9	C1—Pd1—N3^i^	94.81 (9)
O1—C12—N3	126.6 (2)	N3—Pd1—N3^i^	179.999 (1)
O1—C12—C11	116.8 (2)	C19—O2—H1	109.1
			
N1—C2—C3—N2	−0.1 (3)	C12—C11—N1—C1	57.6 (3)
N2—C4—C5—C10	140.4 (2)	C12—C11—N1—C2	−116.4 (3)
N2—C4—C5—C6	−40.1 (3)	N1—C1—N2—C3	1.2 (3)
C10—C5—C6—C7	0.5 (4)	Pd1—C1—N2—C3	−168.1 (2)
C4—C5—C6—C7	−179.0 (2)	N1—C1—N2—C4	175.0 (2)
C5—C6—C7—C8	−0.4 (4)	Pd1—C1—N2—C4	5.7 (4)
C6—C7—C8—C9	0.0 (5)	C2—C3—N2—C1	−0.7 (3)
C7—C8—C9—C10	0.4 (5)	C2—C3—N2—C4	−174.4 (2)
C6—C5—C10—C9	−0.1 (4)	C5—C4—N2—C1	−71.5 (3)
C4—C5—C10—C9	179.4 (2)	C5—C4—N2—C3	101.4 (3)
C8—C9—C10—C5	−0.4 (4)	O1—C12—N3—C13	−16.9 (4)
N1—C11—C12—O1	136.3 (2)	C11—C12—N3—C13	161.5 (2)
N1—C11—C12—N3	−42.3 (3)	O1—C12—N3—Pd1	162.1 (2)
C18—C13—C14—C15	−1.1 (4)	C11—C12—N3—Pd1	−19.5 (3)
N3—C13—C14—C15	177.1 (2)	C18—C13—N3—C12	30.0 (4)
C13—C14—C15—C16	−0.5 (4)	C14—C13—N3—C12	−148.0 (2)
C14—C15—C16—C17	1.3 (4)	C18—C13—N3—Pd1	−149.0 (2)
C15—C16—C17—C18	−0.5 (4)	C14—C13—N3—Pd1	32.9 (3)
C16—C17—C18—C13	−1.1 (4)	N1—C1—Pd1—N3	−40.62 (19)
C14—C13—C18—C17	1.9 (4)	N2—C1—Pd1—N3	127.6 (3)
N3—C13—C18—C17	−176.1 (2)	N1—C1—Pd1—N3^i^	139.37 (19)
N2—C1—N1—C2	−1.3 (3)	N2—C1—Pd1—N3^i^	−52.4 (3)
Pd1—C1—N1—C2	170.12 (17)	C12—N3—Pd1—C1^i^	−126.10 (19)
N2—C1—N1—C11	−176.1 (2)	C13—N3—Pd1—C1^i^	52.97 (18)
Pd1—C1—N1—C11	−4.7 (3)	C12—N3—Pd1—C1	53.90 (19)
C3—C2—N1—C1	0.9 (3)	C13—N3—Pd1—C1	−127.03 (18)
C3—C2—N1—C11	175.4 (2)		

Symmetry codes: (i) −*x*+1, −*y*+1, −*z*+2.

### Hydrogen-bond geometry (Å, °)

**Table:**

*D*—H···*A*	*D*—H	H···*A*	*D*···*A*	*D*—H···*A*
O2—H1···O1^ii^	0.92	1.81	2.727 (3)	172
C2—H2···O1^iii^	0.95	2.36	3.232 (3)	152
C18—H18···O1	0.95	2.32	2.842 (3)	114

Symmetry codes: (ii) *x*, −*y*+1/2, *z*−1/2; (iii) −*x*+1/2, *y*−1/2, *z*.
